# Supplementary material for: A Novel and Automated Approach to Classify Radiation Induced Lung Tissue Damage on CT Scans
Source: Cancers (Basel). 2022 Mar 5;14(5):1341. doi: 10.3390/cancers14051341 (PMC8909378; doi:10.3390/cancers14051341)
Supplement: Supplementary file 1 [file cancers-14-01341-s001.zip › cancers-1547141-supplementary.pdf]

Supplementary Materials

Convolutional Neural Networks

In our study we used 2D UNet networks to perform multiclass segmentation of five distinct tissue classes and the background.

Preliminary investigations indicated potential challenges originating for a severe class imbalance. We conducted a set of initial experiments to determine the optimal approach, testing a full slice (384x288 pixels) against a patch-based approach (a patch of 64x64 pixels), both using Unet architectures In the patch-based approach we directly mitigated for the class imbalance creating a training dataset with patches divided based on the prevalence of lung tissue class represented in a patch and then oversampling from under-represented classes to balance the differences. From our initial experiments the patch-based approach showed lower performance than a full CT slice approach, thus we opted for the latter throughout the rest of our studies. We also tested a five downsampling stages configuration for a full slice approach, but we did not observ improvements over the four downsampling counterpart.

Our chosen network was a 2D UNet, with four down-sampling stages. We tested batch and instance normalization, seeing marginally better performance during our initial experiments for instance normalization. We used different configurations of number of filters at the initial layer (32 and 64) and loss functions (Weighted Cross Entropy, Lovasz and Dice). A basic configuration of the network with 64 filters in the initial layer is shown in Figure 1.

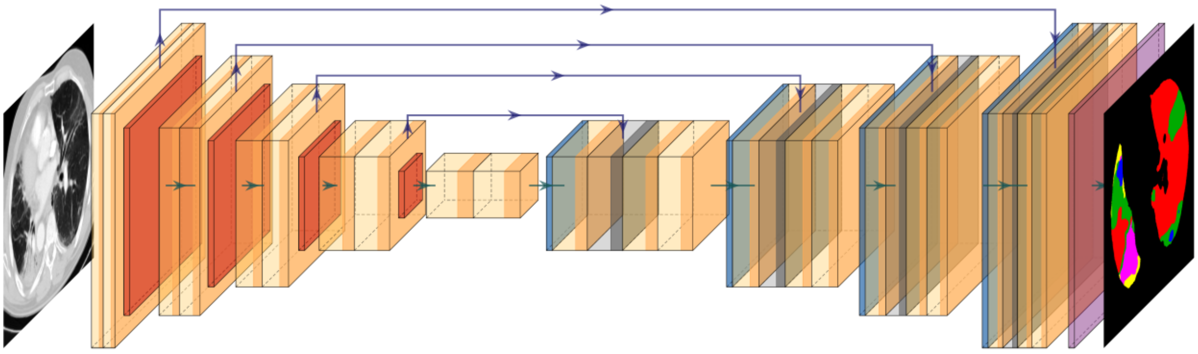

Figure S1. *A schematic description of a single 2D UNET network used as a segmentation method. We present here a version with 64 channels at the initial layer. In our work we used both 64 and 32 filters at the initial layer.*

The networks with different configurations were trained independently as shown in Figure 1, and only combined in an ensemble at the inference stage, where the outputs of the softmax layers were added and subsequently the argmax operation was performed. In this way we could take into account the confidence of particular label by an individual network. That is shown in schematic images in Figure 2 for an ensemble of 3 networks used at stage one ground truth data generation and for 6 networks at stage two ground truth data generation in Figure 3. The configuration with the ensemble of 6 networks was also used as the final method configuration during the inference on the testing dataset.

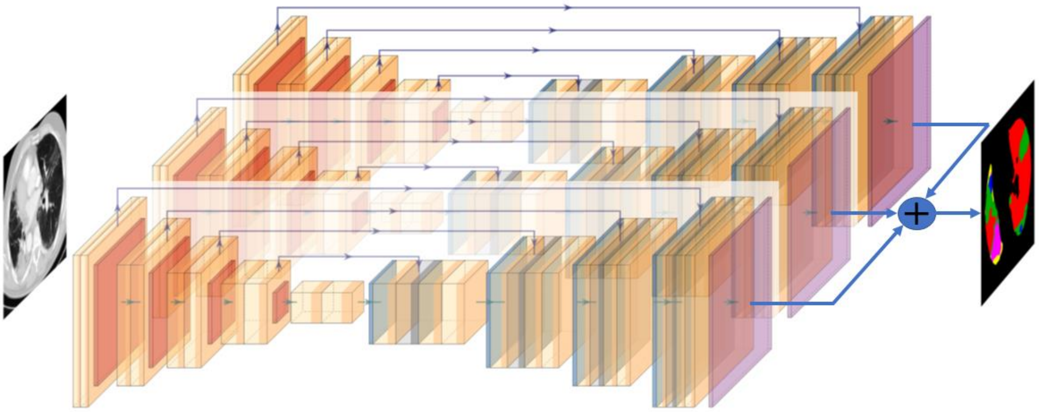

Figure S2. *A schematic description of an ensemble of three networks used in our stage one ground truth data generation method. Prior to combining them in the ensemble they were individually trained as in Figure S1.*

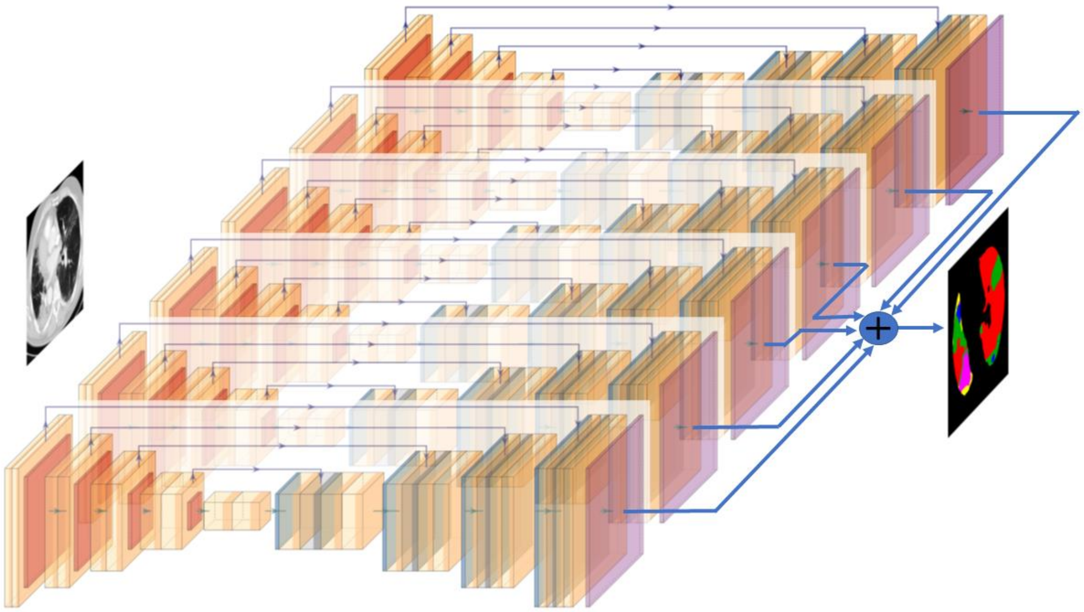

Figure S3. *A schematic description of an ensemble of six networks used in our stage two ground truth data generation method. Prior to combining them in the ensemble they were individually trained as in Figure S1.*

Before presenting CT images to the networks, images were cropped around lung regions to a fixed size of 288x384. If the cropped images extended beyond the fixed size, the images were resized to fully include the lungs with the same scaling parameter used for both axes. The fixed size of 288x384 was chosen as that allowed us to resize only approximately 10% of slices. The image intensities were clipped to lung intensity window [-1000, 300] and rescaled to [0, 1]. We applied geometric and intensity data augmentation in a form of random left-right and up-down image flipping, random rotations, elastic deformations, and gamma intensity augmentation. The hyperparameters used for training and inference are summarized in Table 1.

Table S1. *Summary of parameters used for training and inference of the CNNs.*

Parameter	Value/setting
Architecture	2D Unet
Input image size	288 × 384
Number of down/up sampling stages	4
Number of filters in initial layer	32 or 64
Number of filters in bottleneck layer	512 or 1024
Batch size	8
Loss Function	Weighted Cross Entropy, Lovasz or Dice
Validation metric	Dice
Normalisation layer	Instance normalisation
Activation function	ReLu
Downsampling function	MaxPooling
Upsampling function	BiLinear upsampling
Final activation function	SoftMax
Maximum number of Epochs	200
Optimisation method	Adam
Optimisation parameters	Betas = (0.9, 0.999), eps = 1e-8, weight decay = 0.0005
Initial learning rate	0.001
Learning rate scheduler	Reduce when reaches plateau, patience = 10, factor = 0.1
CT images intensity cropping	[-1000, 300]
Input image intensity scale	[0, 1]
Mean image intensity	0.2
STD image intensity	0.19
Intensity data augmentation	Prob = 0.5, gamma = [0.8, 1.2]
Elastic data augmentation	Prob = 0.5, spacing = (100, 100), magnitude = [5, 5]
Rotation data augmentation	Prob = 0.5, angle = [-30, 30]
Flip left-right	Prob = 0.5
Flip up-down	Prob = 0.5
